# Altered Default Mode and Sensorimotor Network Connectivity With Striatal Subregions in Primary Insomnia: A Resting-State Multi-Band fMRI Study

**DOI:** 10.3389/fnins.2018.00917

**Published:** 2018-12-06

**Authors:** Li Wang, Kun Wang, Jiang-Hong Liu, Yu-Ping Wang

**Affiliations:** ^1^Department of Neurology, Xuanwu Hospital, Capital Medical University, Beijing, China; ^2^Beijing Puren Hospital, Beijing, China

**Keywords:** primary insomnia, resting state, fMRI, striatum, functional connectivity

## Abstract

**Background:** Primary insomnia is a high prevalent sleep disorder. Disturbed brain activity during reward, emotional, and cognitive processing have been observed in insomnia patients. Studies have implicated a critical role of the striatum in these dysfunctions. However, there have been no direct investigations on the whole-brain functional connectivity (FC) of the striatum in insomnia.

**Methods:** We analyzed the group differences in the FC images of 6 predefined striatal subregions based on the multi-band resting-state fMRI data of 18 insomnia patients and 16 healthy controls.

**Results:** We found increased positive FC in the bilateral medial frontal gyrus for bilateral dorsal caudate (DC) and left inferior ventral striatum (VS) subregions, but increased negative FC in the bilateral inferior parietal lobe for the left inferior VSi and right dorsal caudal putamen (DCP) subregions, and in the lateral temporal, occipital, and primary sensorimotor areas for the bilateral DC and left superior VS subregions. The FC between the right DCP and right inferior parietal lobe showed significant positive correlation with Pittsburgh Sleep Quality Index (PSQI).

**Conclusion:** The findings indicate disturbed striatal FC with the default mode network (DMN), the visual and somatosensory areas in insomnia, which likely reflects an inappropriate reward or emotional significance attribute to self-reflection, episodic memory, sensory-perception processes. The altered striatal FC might increase the risk of insomnia patients to develop depression and anxiety.

## Introduction

Insomnia disorder is a highly prevalent illness afflicting 10 to 20% of the adult population worldwide (Kraus and Rabin, 2012; Buysse, 2013). By affecting a series of reward, emotional and cognitive functions, insomnia confers an increased risk for a variety of psychiatric disorders, especially for depression and anxiety (Durmer and Dinges, 2005; Morin and Benca, 2012). Although the pharmacological and behavioral therapies have been efficient for insomnia, the drugs has potential risk of dependency, and the behavior therapies are inconvenient and time consuming. The development of more safer or practical alternatives for insomnia required more exact understanding of its neural mechanism.

Insomnia have been associated with disturbances of several psychopathological dimensions involving reward, emotional, and cognitive executive functions (Van Someren et al., 2015; Medrano-Martínez and Ramos-Platón, 2016), which have been also implicated in depression and anxiety (Ninan and Berger, 2001; Okamoto, 2009). The striatum is a central region of reward system. It connects to multiple cortical and subcortical regions and constitute a closed cortical-BG-thalamo-cortical loop important for widespread emotional and cognitive functions (Parent and Hazrati, 1995; Draganski et al., 2008). This unique feature makes the striatum an important entry point for investigating the neural mechanism of insomnia.

With the help of functional magnetic resonance imaging (fMRI), past researches have significantly improved our understanding of the neural circuitry of insomnia. While direct examinations of the striatal functional connectivity (FC) are limited, insomnia-related abnormalities have been detected in the striatum and its projection areas. For instance, those who have an experience of SD exhibited increased ventral striatum (VS) and ventromedial prefrontal cortex (vmPFC) activation during the receipt of reward stimuli (Venkatraman et al., 2007, 2011). In insomnia, researchers observed reduced recruitment of the left caudate during executive functioning, which was driven by attenuated input from a projecting vmPFC area (Stoffers et al., 2014). Others found an inability to modulate the DMN activation during working memory performance (Drummond et al., 2013) and impaired structural covariance between the anterior and posterior areas of the DMN (Suh et al., 2016). Interestingly, most of these regions fall within the reward system and default mode network (DMN), a network that is involved in self-reflection and affective cognition (Habeck et al., 2012; Hamilton et al., 2015; Mohan et al., 2016). According to the hyper-arousal theory (Kalmbach et al., 2018), the loss of sleep has been also associated with a sensory and cortical hyper-excitability. Consistently, the resting-state fMRI (R-fMRI) studies exhibited insomnia-related changes (indexed by amplitude of low-frequency fluctuation (ALFF) or seed-based FC) in the sensorimotor-related areas involving the lateral temporal and occipital lobes, and primary sensorimotor cortex (Killgore et al., 2013; Li C. et al., 2016; Zhou et al., 2016; Ran et al., 2017).

However, these investigations primarily focused on task-induced responses in the striatum and its projection regions, which provides a limited window into changes in whole-brain FC of insomnia patients, especially for a high heterogeneous structure like the striatum. Of note, the striatum consists of 3 spatially non-overlapping areas: the caudate, VS, and putamen (De Havas et al., 2012). The VS receives projections from the vmPFC and limbic structures. The dorsal caudate (DC) receives projections from the dorsolateral PFC, while the dorsal putamen receives projections from the primary sensorimotor areas. The R-fMRI provides a powerful tool to systematically characterize the FC in a high heterogeneous region and has been successfully used to map the RSFC of the striatal subregions in normal (Di Martino et al., 2008) and pathological (Di Martino et al., 2011; Gabbay et al., 2013) populations, yet the existing R-fMRI studies have not examined the whole-brain FC of the striatum in insomnia patients.

Therefore, we conducted this study to examine the striatal FC in 18 insomnia patients and 16 healthy controls (HCs) based on their multi-band R-fMRI data. We collected the R-fMRI data with a sampling rate of 0.75 s, which is higher than the traditional 2–2.5 s. A high sampling rate was thought to provide more exact temporal information on functional integration among brain regions and reduce the effects of high-frequency physiological noise (Liao et al., 2013). Seed-based analysis was used to interrogate the FC patterns of 6 striatal subregions defined *a priori* (Di Martino et al., 2008). Based on the behavioral and neuroimaging findings noted above, we hypothesized that altered FC in the striatal subregions will emerge in the brain regions within the DMN and reward system.

## Materials and Methods

### Subjects

The sample consisted of 18 patients with insomnia and 16 HCs. All subjects were 20–60 years old and right-handed. Those with insomnia were recruited from Beijing Xuanwu Hospital or local advertisements. The diagnosis was made by clinical interview conducted by a psychiatrist according to the DSM-IV criteria. Patients were required to have a complaint of difficulty in falling asleep, maintaining sleep, or early awakening, lasting at least one month. The sleep symptoms were not secondary to any medications, substance abuse, physical, or psychiatric disorders. Since depression and anxiety are often accomplished by insomnia, we required that patients could have related symptoms, but they needed to have scores of 17-item Hamilton Rating Scale for Depression (HAMD) < 14 and Hamilton Anxiety Rating Scale (HAMA) < 14. Among the 18 patients, 10 have a history of psychotropic medication, but all patients have no use of any medications for at least one month prior to the study. The sample characterizes were provided in Table 1.

**Table 1:** Sample characteristics.

Variables	Insomnia (n = 18)	Control (n = 16)	Statistics	
			*t*	*p*
Age (years)	21.9 ± 3.3	22.4 ± 3.1	–0.846	0.4
Gender (male/female)	5/13	6/10	0.366	0.717
Education (years)	13.9 ± 2.2	14.2 ± 1.7	–0.831	0.408
FD	0.1 ± 0.0	0.1 ± 0.04	1.279	0.204
PSQI	17.3 ± 1.6	1.3 ± 1.7	28.208	< 0.001
HAMD-17	8.6 ± 4.0	1.8 ± 2.0	6.100	< 0.001
HAMA	13.8 ± 5.8	1.3 ± 1.5	8.320	< 0.001


*Data are presented as mean ± SD. FD, Framewise displacement; PSQI, Pittsburgh Sleep Quality Index; HAMD, Hamilton Rating Scale for Depression; HAMA, Hamilton Anxiety Rating Scale.*

All subjects have no history of neurological diseases and current unstable medical conditions. Control subjects have no any sleep complaints or psychiatric disorders. This study followed the guidelines of the Declaration of Helsinki and was approved by the Institutional Review Board of Beijing Xuanwu Hospital. All subjects signed written informed consent. All subjects completed the Pittsburgh Sleep Quality Index (PSQI) (Buysse et al., 1989), 17-item HAMD (Riskind et al., 1987), and HAMA (Shear et al., 2001).

### MRI Acquisition

Images were acquired with a Siemens 3.0 Tesla scanner. The resting-state functional images were obtained by using a multi-band echo-planar imaging sequence with the following parameters: repetition time (TR)/echo time (TE), 750 ms/30 ms; 90° flip angle; thickness/gap, 3 mm/0 mm; 48 slices; 7 min. For a registration propose, T1-weighted structural images were obtained using a magnetization-prepared rapidly acquired gradient-echo (MPRAGE) sequence: TR/TE, 2300 ms/3.01 ms; thickness/gap, 1.0/0 mm; matrix, 256 × 256; voxel size, 1 × 1 × 1 mm^3^; 9° flip angle. Before the resting-state scans, subjects were instructed to keep their eyes closed, remain still without head movement, not think of anything in particular, and not fall asleep during the scan. All subjects reported a good adherence to these instructions through confirmation immediately after the scans. No subjects showed obvious structural damage based on their conventional MRI scans.

### Data Preprocessing

The R-fMRI data were preprocessed with Data Processing Assistant for R-fMRI (DPARSF)^1^ based on Statistical Parametric Mapping (SPM12).^2^ After removing the first ten volumes, the remaining 200 volumes were corrected for different signal acquisition times. The functional volumes were motion-corrected using a six-parameter rigid-body transformation. All subject satisfied the head motion criteria of less than 2 mm maximum displacement in any direction of x, y, and z and 2° in any angular dimension. Then, the nuisance signals (including Friston 24-parameter model (Yan et al., 2013) of head-motion parameters, linear trend, cerebrospinal fluid and white matter signals) were regressed out. Derived images were normalized to Montreal Neurological Institute (MNI) space and re-sampled with a 2 × 2 × 2 mm^3^ resolution using transformation parameters estimated by unified segmentation algorithm (Friston et al., 1996). The transformed images were then band-pass filtered (0.01–0.1 Hz). Given a possible confounding effect of micro-movements on resting-state FC (Power et al., 2012), the framewise displacement (FD) values were computed for each subject using the Jenkinson fomula (Jenkinson et al., 2002) to reflect the temporal derivative of the movement parameters. All subjects satisfied the criterion of mean FD < 0.2 mm.

### Striatal FC

**FIGURE 1:**
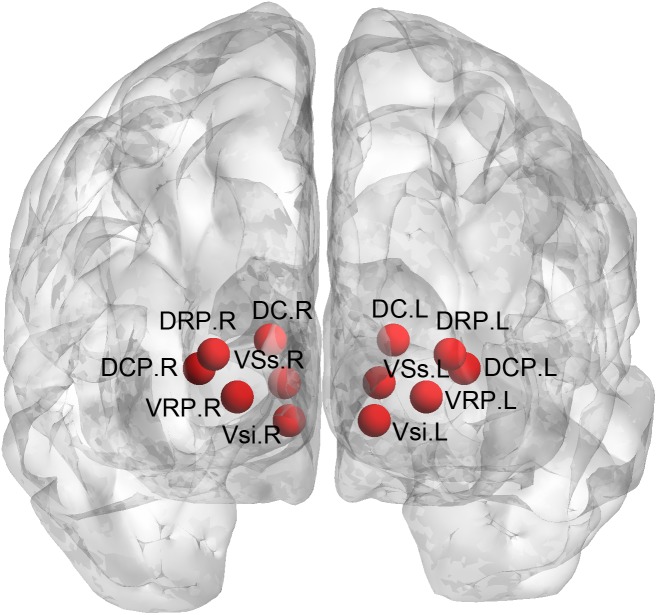
Definition of the striatal subregions. DC, dorsal caudate; VSs, superior ventral striatum; VSi, inferior ventral striatum; DRP, dorsal rostral putamen; DCP, dorsal caudal putamen; VRP, ventral rostral putamen.

We used seed-based approach to compute the FC of the striatal subregions. Specifically, the seeds were defined (MNI152 space) bilaterally in the DC (*x* = ± 13, *y* = 15, *z* = 9), superior VS (VSs) (*x* = ± 10, *y* = 15, *z* = 0), inferior VS (VSi) (*x* = ± 9, *y* = 9, *z* = -8), dorsal rostral putamen (DRP) (*x* = ± 25, *y* = 8, *z* = 6), dorsal caudal putamen (DCP) (*x* = ± 28, *y* = 1, *z* = 3), and ventral rostral putamen (VRP) (*x* = ± 20, *y* = 12, *z* = -3) (Di Martino et al., 2008), with each region covering 27 voxels in 2 mm^3^ space (radius = 3.5 mm). The placement of these subregions was shown in Figure 1. We extracted the mean time courses of the BOLD signals of each striatal subregion and computed their correlations with the rest of the whole brain. This procedure generated 12 FC images of the striatal subregions (six per hemisphere) for each subject. The r-value correlation images were *z*-value converted, and then, smoothed with a 6-mm Gaussian kernel.

### Statistical Analysis

#### Between-Group Differences

Independent-sample *t*-tests were performed on the FC images of the striatal subregions between insomnia patients and HCs to determine their differences, with age, educational level, and mean FD served as covariates. The results were corrected for multiple comparisons with a combination of cluster *p* < 0.05 and voxel *z*-threshold of 2.3 (which corresponds to *p* < 0.0107) according to Gaussian Random Field (GRF) theory, which lead to a corrected *p* < 0.05. Because we have used the multiple comparison correction for each subregion and dividing 0.005 and 0.05 by 12 would result in very small *p* value and can be too conservative for our exploration, we chose to correct for each subregion but not for the subregion number in these comparisons.

### Relationships Between Striatal FC and Clinical Variables

The partial correlation analysis was performed between the FC values of the clusters showing significant group differences in the striatal FC and clinical variables (including the illness duration and PSQI) within the insomnia patients, with age, educational level, and mean FD served as covariates. The statistical significance was determined by a bonferroni corrected *p* < 0.025 (0.05/number of clinical variable 2). We also performed whole-brain voxel-wise regression analysis between striatal FC images and the PSQI score. Given that this study recruited patients with a wide range of age, i.e., 20–60 years, we performed a correlation analysis between the striatal FC and age, controlling for educational level and mean FD as covariates.

## Results

### Demographic and Clinical Characteristics

As shown in Table 1, there were no significant differences in age, gender distribution, and years of education between insomnia patients and HCs. Patients exhibited significantly higher scores on PSQI, HAMD, and HAMA than HCs.

### Between-Group Differences in Striatal FC

**FIGURE 2:**
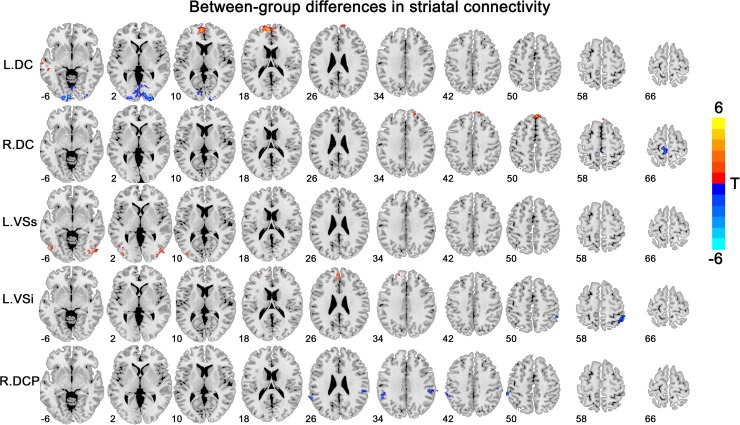
Between-group differences in striatal connectivity. DC, dorsal caudate; VS, ventral striatum; DCP, dorsal caudal putamen. The red colors indicate increased connectivity in primary insomnia patients compared with the healthy controls, while the blue colors indicate contrary connectivity.

Overall, the insomnia patients exhibited altered striatal FC with the DMN [including the vmPFC and inferior parietal lobe (IPL)], the lateral temporal and occipital cortex, and primary sensorimotor areas, with overlapping and distinct changes among different striatal subregions (Figure 2 and Table 2). The details for each subregion were introduced below.

**Table 2:** Between-group differences (insomnia vs. healthy control) in striatal connectivity.

Seed	Region with altered FC	Voxels	Peak MNI coordinate	Peak intensity	Correlation	
					BN	HC
R-DC	Superior medial frontal gyrus	441	–8, 58, 16	5.976	+^∗^	–^∗^
	Middle temporal gyrus	198	–60, –16, –14	5.206	+^∗^	–^∗^
	Calcarine	854	–6, –94, –6	–5.722	–	+
L-DC	Superior medial frontal gyrus	176	2, 52, 46	5.084	+^∗^	–^∗^
	Paracentral lobule	162	–6, –36, 66	–4.527	–^∗^	+^∗^
L-VSs	Middle occipital gyrus	276	48, –76, –10	4.307	+	–^∗^
	Inferior occipital gyrus	219	–36, –66, –12	3.793	+	–^∗^
L-VSi	Medial orbital frontal gyrus	168	–4, 40, –24	4.530	+^∗^	–^∗^
	Superior medial frontal gyrus	167	–18, 52, 38	4.154	+^∗^	–^∗^
	Inferior parietal lobule	142	54, –44, 54	–4.120	–^∗^	–
R-DCP	Inferior parietal lobule +	186	54, –26, 38	–4.041	–^∗^	–
	Inferior parietal lobule	167	–64, –44, 26	–3.789	–^∗^	–


*R, Right; L, Left; DC, dorsal caudate; VSs, ventral striatum superior; VSi, ventral striatum inferior; DCP, dorsal caudal putamen; *t*: *t*-score of peak of connectivity or local maxima.*

#### DC

We observed increased FC [a transition from negative (HCs) toward positive (patients) FC] in the bilateral superior medial frontal gyrus and left middle temporal gyrus for the bilateral DC subregions. A contrary change was observed in the left paracentral lobule (PCL) for the left DC subregion, and in the left calcarine for the right DC subregion.

#### VSs, VSi

The group effect was significant on the left-sided VSs and VSi subregions. Specifically, increased FC [a transition from negative (HCs) toward positive (patients) FC] was observed in the right middle and left inferior occipital cortex for the left VSs subregion and in the left medial OFC for the left VSi subregion, while increased negative FC was observed in the right IPL for the left VSi subregion.

#### DCP, DRP, VRP

**FIGURE 3:**
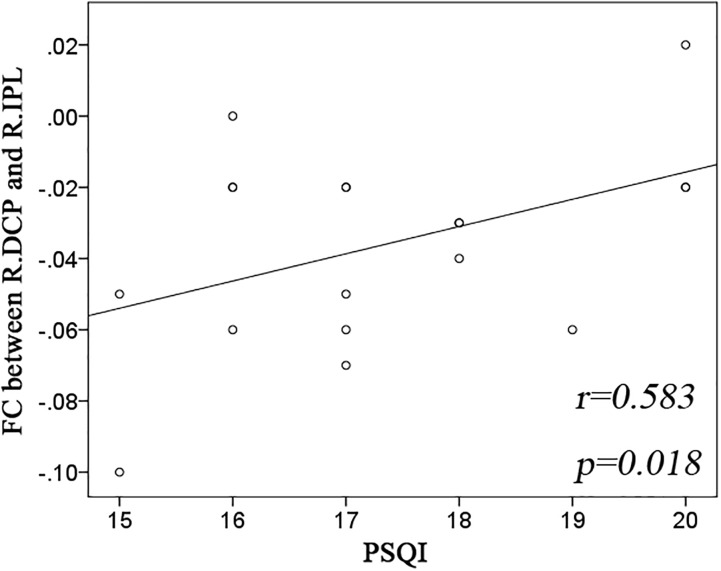
Significant correlation between striatal connectivity and PSQI. Significant positive correlation was observed in the functional connectivity (FC) between the right dorsal caudate putamen (R.DCP) subregion and right inferior parietal lobe (R.IPL) with Pittsburgh Sleep Quality Index (PSQI).

Significant group effect was observed only in the right DCP subregions. The right DCP subregion exhibited increased negative FC with the bilateral IPL, which showed significant negative correlation with the PSQI scores (Figure 3).

The connectivity z scores (mean +/- SD) of clusters with significant group differences were shown in Supplementary Figure S1. In order to state the observed differences are truly only for one specific subregion, we showed the connectivity z scores for other subregions in Supplementary Figure S2. The independent-sample *t*-tests did not show significant between-group differences for these *z* scores. No significant correlations were found between striatal FC and age, between the striatal FC and PSQI score in the whole-brain voxel-wise regression analysis.

## Discussion

This is to our knowledge the first study that systematically examined the whole-brain FC of the striatum in patients with insomnia. We observed altered FC in the DC, VS, and putamen subregions, with both hyper- and hypo-connectivity. Specifically, the changes in striatal FC were primarily distributed over the DMN, the lateral temporal and occipital cortex, and the primary sensorimotor cortex, which is basically consistent with our hypothesis. The right-sided FC between the DCP and IPL showed a good predictive power for the severity of insomnia as assessed by PSQI.

The first important finding of this study is altered striatal FC (both increase and decrease) in the anterior and posterior components of DMN. The FC between the right DCP subregion and right IPL could be a potential predictor for insomnia severity. DMN is highly activated at rest but deactivated during goal-directed cognitive tasks (Buckner et al., 2008). An enormous body of literature suggests that the DMN could be divided into anterior and posterior divisions (Kim and Lee, 2011; Mak et al., 2017). The anterior DMN primarily includes the vmPFC and is involved in self-reflective thought, while the posterior DMN includes the IPL and precuneus, and is more associated with episodic memory (Zhu et al., 2012). The maintenance of healthy sleep, emotion, and cognition is dependent on appropriate activity within the DMN and balance between its anterior and posterior divisions (Horovitz et al., 2009), which has been disturbed under the condition of sleep loss. For instance, the controlled SD in healthy persons has induced an aberrant activity both within the DMN and between the DMN and its negatively correlated regions (De Havas et al., 2012; Dai et al., 2015). An inability to inhibit the DMN and engage task-appropriate brain regions was observed in insomnia patients during working memory performance (Drummond et al., 2013). Further, insomnia patients showed disrupted FC within the DMN subregions at rest (Nie et al., 2015; Yu et al., 2018).

The preliminary evidence indicates a FC disturbance between the DMN and striatum in insomnia. It was found that acute SD induced increased response in the vmPFC to reward stimuli (Venkatraman et al., 2007, 2011). The caudate has been less recruited when insomnia patients performing cognitive executive task, driven by an attenuated input from the left mid-posterior OFC (Stoffers et al., 2014). Moreover, studies have shown decreased FC between the precuneus and bilateral caudate (Bluhm et al., 2009) in MDD but increased FC between the medial PFC and VS in subthreshold depression (Hwang et al., 2016), suggesting a disparate change in striatal FC with the anterior and posterior DMN in depressed individuals. This phenomenon seems to be similar to our findings that indicate an abnormal transition toward positive FC in the bilateral medial frontal gyrus, but strengthened negative FC in the bilateral IPL in insomnia patients, suggesting that the pathological mechanisms of depression and insomnia have something in common. Given a basic role of the DMN in sleep, emotion, and cognition, and its importance in the pathophysiology of depression and anxiety (Disner et al., 2011; Bas-Hoogendam et al., 2016), we considered that disturbed DMN-striatum FC likely indicates an inappropriate reward evaluation on self-referential, affective cognition, and emotion regulatory processes, which promote emotional and cognitive impairments and increase the susceptibility of depression or anxiety disorders.

Another important finding is altered striatal FC in the visual and somatosensory areas involving the lateral temporal and occipital cortex, and PCL. According to the hyperarousal theory (Palagini et al., 2018), insomnia occurs as a result of somatic and cognitive hyper-arousal caused by active stressors and cognitive rumination. Congruently, increased brain activity (indexed by ALFF) in the primary sensorimotor, the temporal and occipital cortex was observed in those with insomnia or an experience of SD (Dai et al., 2016; Li C. et al., 2016; Zhou et al., 2016; Ran et al., 2017). From a connectivity perspective, insomnia patients displayed enhanced RSFC among various sensory cortices and supplementary motor area (Killgore et al., 2013) and nodal centrality in the right PCL (Li et al., 2018), which predicted sleep initiation difficulty evaluated with PSQI. By selecting the amygdala as a seed region, researchers observed increased RSFC in the primary sensorimotor cortex of insomnia patients (Huang et al., 2012). The innovation of our research is to relate the functional abnormalities of these visual and somatosensory areas to core region of reward system - the striatum. This imbalanced relationship likely indicates a disturbed reward sensitivity to sensory and perception stimuli, which ultimately disturb the sleep.

The left and right striatal subregions exhibited some common but distinct changes, which intuitively indicates a hemispheric functional asymmetry in insomnia. In support of our proposal, an asymmetric EEG coherence has been observed in patients with insomnia (Kovrov et al., 2006). The diffusion tensor imaging study demonstrated insomnia-related reduction in fractional anisotropy of the body of the corpus callosum (Li S. et al., 2016), which connects two hemispheres and transmits information between the left- and right-sided brain regions. More directly related to our study is the impairments in inter-hemispheric functional incoordination in insomnia patients (Li et al., 2017; Zhou et al., 2018). Therefore, differential changes in striatal FC between two hemispheres could be an important aspect of the pathophysiology of insomnia, which might be another mechanism underlying emotional and cognitive deficits of insomnia patients.

The primary contribution of this study is to provide direct evidence of whole-brain FC changes of the striatal subregions in insomnia. Several issues need to be further addressed. The results were obtained in a small sample of insomnia patients, which should be replicated in more patients in future studies. The disorder specificity of the current results for insomnia remains to be clarified, given that aberrant striatal FC have also been observed in patients with other psychiatric and neurological disorders (Sakai et al., 2011; Stoddard et al., 2016; Huang et al., 2018). Though insomnia-related changes have been reported in the striatal dopamine 2 receptor binding (Broft et al., 2012) and serotonergic neurotransmission in cortical areas (Tiihonen et al., 2004), the molecular basis of the striatal FC changes observed in our study is unclear. A synchronous positron emission computed tomography and fMRI scans is a promising pathway to clarify their associations. Finally, a longitudinal follow-up study with a larger sample will help to confirm our speculation on the relationships among insomnia, striatal FC, and occurrence of depression or anxiety disorders.

## Conclusion

Our findings indicate altered striatal FC with the DMN, the visual and somatosensory networks of insomnia patients. While the functional abnormalities in the DMN, the visual and somatosensory networks have been reported in insomnia patients, to our knowledge, that specific FC changes with the striatal subregions is novel. These FC alteration likely affect the reward evaluation during self-reflective, emotional cognition, sensory and perception processing, which confers a high vulnerability of insomnia patients to develop depressive or anxiety disorders. We hope this will encourage further investigations of new treatment approaches (e.g., brain stimulation technology) for insomnia by normalizing the functional disturbances of striatal connectivity.

## Author Contributions

LW designed and performed the experiments, analyzed the results, and wrote the manuscript. J-HL and KW contributed to clinical data collection and assessment. Y-PW provided guidance for this study. All authors approved the final manuscript.

## Conflict of Interest Statement

The authors declare that the research was conducted in the absence of any commercial or financial relationships that could be construed as a potential conflict of interest.

## Footnotes

1 http://rfmri.org/DPARSF

2 http://www.fil.ion.ucl.ac.uk/spm
