# Supplementary material for: Altered Default Mode and Sensorimotor Network Connectivity With Striatal Subregions in Primary Insomnia: A Resting-State Multi-Band fMRI Study
Source: Front Neurosci. 2018 Dec 6;12:917. doi: 10.3389/fnins.2018.00917 (PMC6291517; doi:10.3389/fnins.2018.00917)
Supplement: Supplementary file 1 [file Data_Sheet_1.docx]

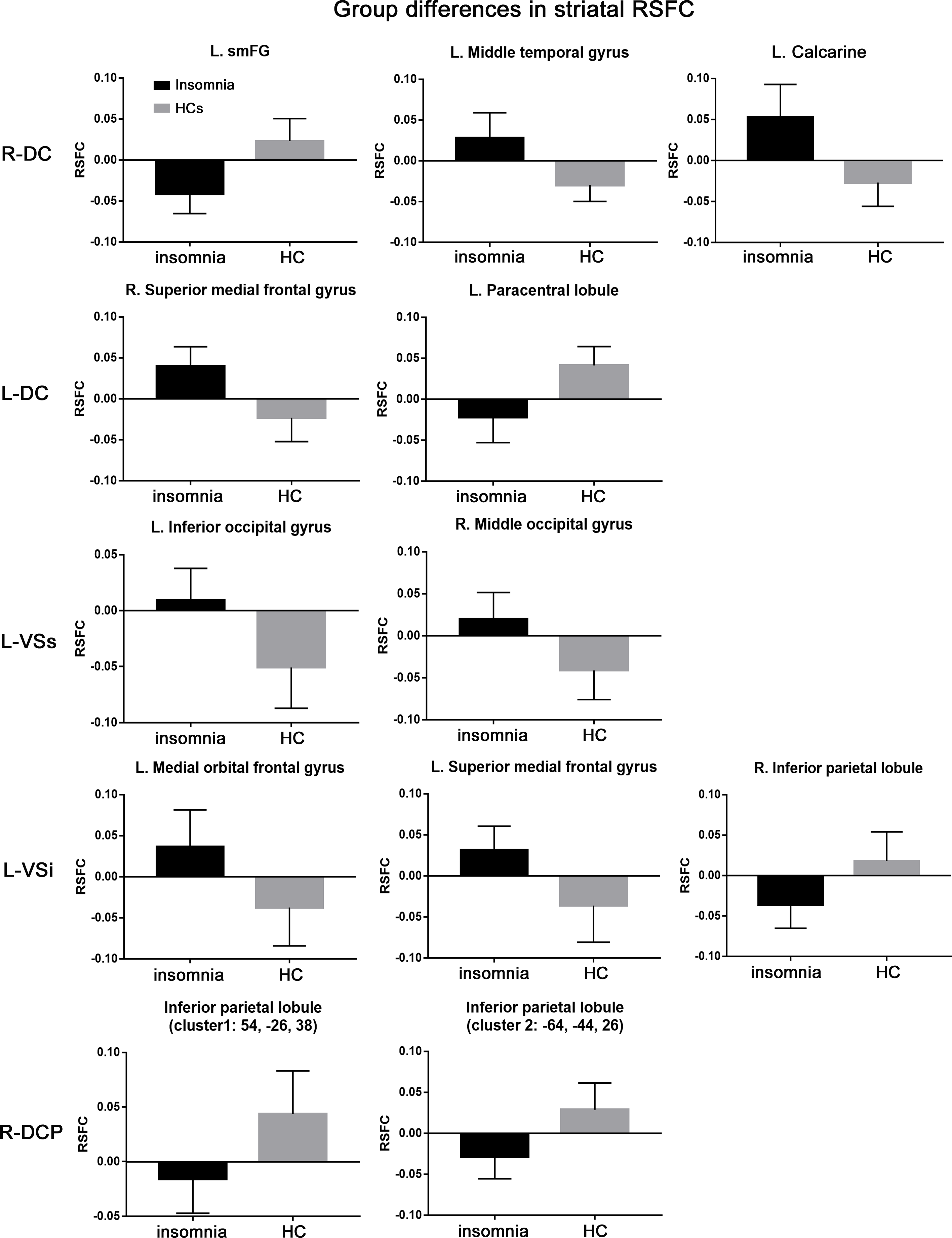


**Figure 1. Between-group differences in resting-state functional connectivity (RSFC) in striatal subregions.**

The images displayed between-group differences in resting-state functional connectivity (RSFC) values in striatal subregions. DC, dorsal caudate; VSs, superior ventral striatum; VSi, inferior ventral striatum; DCP, dorsal caudal putamen. L, left; R, right. HC, healthy control.


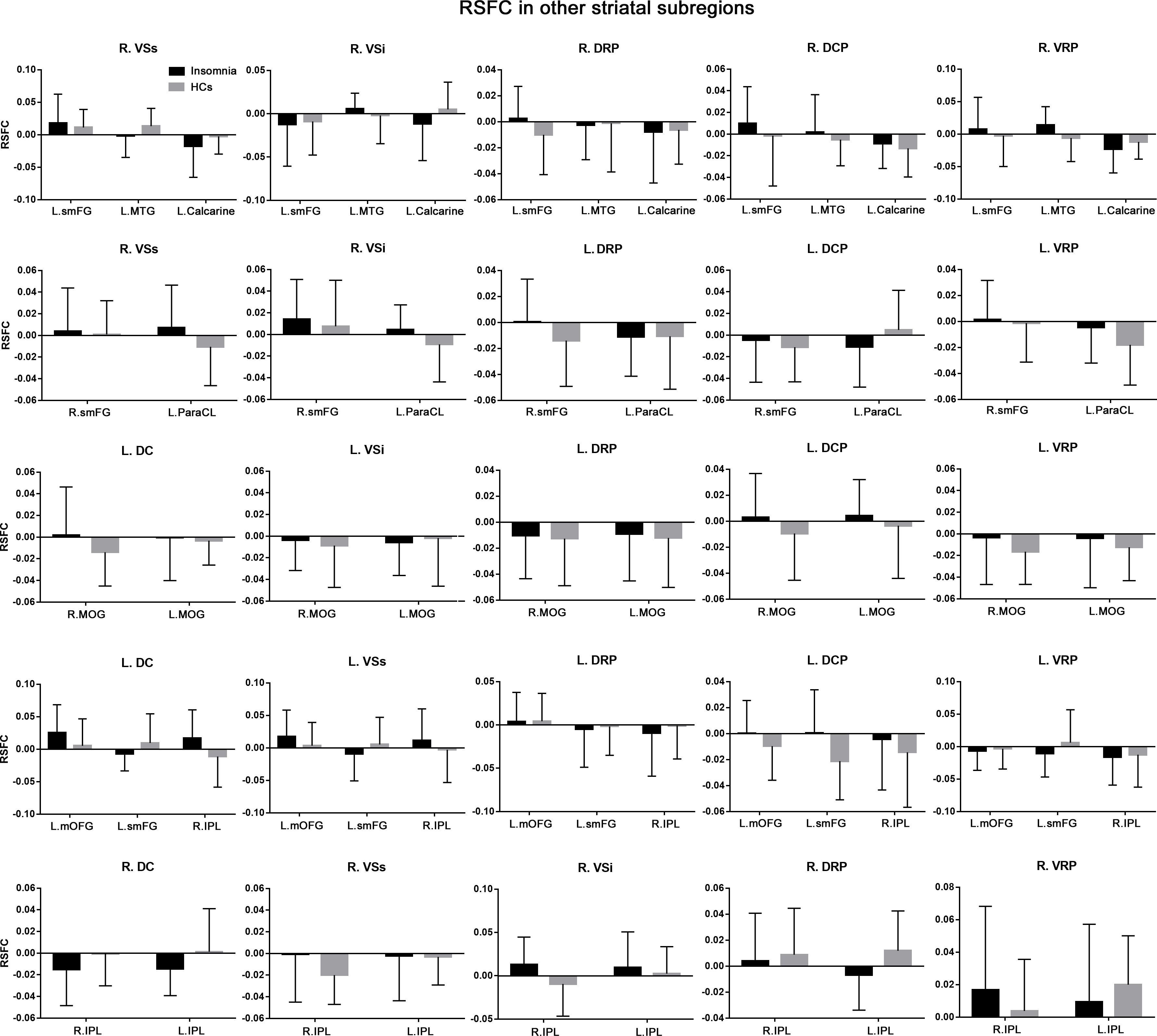


**Figure 2. RSFC in other striatal subregions.**

The images displayed the corresponding resting-state functional connectivity (RSFC) values in other subregions showing between-group differences in each striatal subregion. DC, dorsal caudate; VSs, superior ventral striatum; VSi, inferior ventral striatum; DRP, dorsal rostral putamen; DCP, dorsal caudal putamen; VRP, ventral rostral putamen. smFG, superior medial frontal gyrus; MTG, middle temporal gyrus; ParaCL, paracentral lobule; MOG, middle occipital gyrus; IOG, inferior occipital gyrus; mOFG, medial orbital frontal gyrus; IPL, inferior parietal lobule. L, left; R, right. HC, healthy control.
